# Survival of TNF antagonists in spondylarthritis is better than in rheumatoid arthritis. Data from the Spanish registry BIOBADASER

**DOI:** 10.1186/ar1941

**Published:** 2006-04-18

**Authors:** Loreto Carmona, Juan J Gómez-Reino

**Affiliations:** 1 Research Unit, Spanish Society of Rheumatology, Madrid, Spain Marques de Duero 5, 28001 Madrid, Spain; 2 Rheumatology Service and Department of Medicine, Hospital Clínico Universitario, Medical School, Universidad de Santiago de Compostela, Santiago de Compostela, A Choupana s/n, 15706 SantiagoSpain

## Abstract

The aim of the present work is to compare drug survival and safety of infliximab, etanercept, and adalimumab (tumor necrosis factor [TNF] antagonists) in spondylarthritis (SpA) with those of rheumatoid arthritis (RA). To this purpose, we analysed the data in BIOBADASER (2000–2005), a drug registry launched in 2000 for long-term follow-up of the safety of these biologics in rheumatic diseases. The rates of drug discontinuation and adverse events (AEs) in SpA (*n *= 1,524) were estimated and compared with those of RA (*n *= 4,006). Cox regression analyses were used to adjust for independent factors. Total exposure to TNF antagonists for SpA was 2,430 patient-years and 7,865 for RA. Drug survival in SpA was significantly greater than in RA at 1, 2, and 3 years. The hazard ratio (HR) for discontinuation in SpA compared with RA was 0.66 (95% confidence interval [CI], 0.57–0.76) after adjustment for age, gender, and use of infliximab. The difference remained after controlling for the individual medication and its place in the sequence of treatment. There were fewer SpA patients with AEs (17%) than RA patients (26%; *p *< 0.001). The HR for AEs in SpA was 0.80 (95% CI, 0.70–0.91) compared with RA after adjustment for age, disease duration, and use of infliximab. In conclusion, due in part to a better safety profile, survival of TNF antagonists in SpA is better than in RA. TNF antagonists are at present a safe and effective therapeutic option for long-term treatment of patients with SpA failing to respond to traditional drugs. Because chronic therapy is necessary, continual review of this issue is necessary.

## Introduction

The term spondylarthritis (SpA) refers to a group of conditions with inflammation at the entheses, axial skeleton, peripheral joints, and non-articular structures [1-3]. It includes ankylosing spondylitis (AS), reactive arthritis, undifferentiated SpA, juvenile spondylitis, and the arthritis associated with psoriasis or inflammatory bowel diseases. These conditions occur in approximately 1% of the general population [3]. Because of overlapping clinical features, diagnosis of any single one from among the several within the group is sometimes difficult. Nevertheless, treatment does not differ very much among the different conditions. Non-steroidal anti-inflammatory drugs (NSAIDs) have a role in symptom modification and disease control in patients with AS [4,5] as do methotrexate and sulfasalazine with psoriatic arthritis (PsA) and AS [6-17]. In both conditions, these drugs have demonstrated some benefit in peripheral arthritis. In axial disease, evidence is lacking.

Recently, tumor necrosis factor (TNF) inhibitors have been found to be safe and effective in the short-term management of AS, PsA, enteropathic arthritis, and juvenile SpA in patients failing to respond to traditional therapies [17-34]. Unlike in rheumatoid arthritis (RA), however, their long-term efficacy and safety in such conditions are largely unknown.

In February 2000, the Spanish Society of Rheumatology (SER) launched a drug registry (BIOBADASER) of patients with any rheumatic condition treated with biologic disease modifiers. In the past 5 years, more than 5,000 patients from 100 centres have been included in the registry and followed up with [35]. Although the emphasis of BIOBADASER is in drug safety, information on drug discontinuation for any cause is gathered as well. For prescription of any biological disease modifier in a context of universal health coverage in Spain, the physician commits himself to assess effectiveness and safety regularly and discontinue medication when appropriate to meet our current guidelines. Thus, drug survival in this particular clinical setting may be considered a surrogate for effectiveness. Consistency of the data in our registry, which have been externally assessed as described in Materials and methods, and comparison of drug survival in different conditions offer a unique opportunity for the detection of relevant differences in safety and effectiveness. In the present work, we describe the differences in the survival and safety of TNF antagonist in SpA compared with the well-known profile in RA.

## Materials and methods

A description of BIOBADASER has been published elsewhere [28], and its protocol and periodical reports are available on its Web page [36]. In brief, BIOBADASER is a drug registry established in February 2000 for active long-term follow-up of rheumatic patients being treated with biological response modifiers. Patients treated with infliximab before the start of the registry were also included if complete history of treatment and information on adverse events (AEs) were available. The registry, which is supported by the SER and funded in part by the Spanish Agency for Medicines and Health-Service Products (Agencia Española de Medicamentos y Productos Sanitarios), notes relevant AEs (RAEs) occurring during treatment. All hospital and community-based Rheumatology Units in Spain were invited to participate in setting up the project. Participation is voluntary, covering approximately 60% of the patients treated with these therapies for rheumatic diseases in Spain. The large number of participating units (100) ensures a true mix of hospital and community-based practices.

A random code is assigned to every patient entered. This code will be kept throughout the follow-up, until death, or until the study closure date. The registry protocol and methods were approved by the Spanish Medicines Agency (Ministerio de Sanidad y Consumo), and the information regarding patients was gathered in the registry and handled according to current official regulations on data protection.

Data collected systematically include gender, date of birth, diagnosis, date of diagnosis, treatment type, date of start, and date of discontinuation. Should a patient discontinue treatment, the main reason for end is also recorded (for example, inefficacy, AE, or other causes). When a patient has an RAE, additional data are registered; these data include the date of occurrence, type, and classification of event according to the World Health Organization Adverse Reaction Dictionary.

Completeness and agreement of data with patient charts were assessed *in situ *by audits of 15% of patients' records between December 2003 and March 2004 and by telephone of 10% of patients' records between December 2004 and January 2005. Non-communication of the TNF antagonist discontinuation was detected in 6% of cases and non-communication of RAE in 16%; non-communication of either discontinuation or RAE was present in 18% of the sample. All errors were corrected accordingly and yielded an expected error rate, in the whole BIOBADASER sample, of approximately 11%.

Guidelines for the use of TNF antagonists in patients with SpA were available through the SER at the beginning of 2005. Criteria for eligibility of patients with axial disease comprise failure to respond to two NSAIDs, bath AS disease activity index (BASDAI) greater than 4, and additional clinical criteria. For peripheral joint disease, TNF antagonists are recommended for patients who failed to respond to two NSAIDs and sulphasalazine and who have a self-assessment of the disease in a visual analog scale greater than 4 cm and an elevation of acute-phase reactants. These guidelines do not contain recommendations for discontinuation. Prior to this date, biologics were used based on the judgement of the rheumatologist, most often following the criteria settled previously for their application as compassionate medication. These criteria were more stringent and include a BASDAI greater than 6.5 units for spinal disease, more than five inflamed joints, and radiographic erosive changes for peripheral arthritis in any case in which a patient failed to respond to two NSAIDs and two disease-modifying antirheumatic drugs (DMARDs). In Spain, infliximab was available for clinical use in August 1999, etanercept in April 2003, and adalimumab in February 2004. For AS, infliximab was approved in May 2003 and etanercept in January 2004. For PsA, infliximab was available in September 2004 and etanercept in December 2002. For the other types of SpA, biologics are used as compassionate medication. All three biologics (infliximab, etanercept, and adalimumab) are provided free of charge to patients and without restriction at the hospital level by the National Health Service.

Data in the present study correspond to those gathered from the start of the registry to February 2005. Data that are incomplete are censored at the time the last reliable information was recorded. The censoring was done to avoid having long drug survival that reflected under-reporting rather than true drug retention.

### Statistical methods

The cumulative rate of discontinuation was calculated using the actuarial method, accounting for multiple failures per patient and for the calendar year. The log-rank test was used to compare the different types of SpA and to compare the survival curves of SpA and RA. Cox regression models were used to measure the differences in discontinuation rate between the two diagnostic groups as well as to measure association between risk factors and discontinuation. Besides estimation of drug survival and retention rate, the incidence rates of AEs were compared, and Cox regression analyses were used to detect and adjust for independent factors other than the diagnostic group. All analyses were performed using the Stata 9.1 program (StataCorp LP, College Station, TX, USA).

## Results

A majority of the patients registered in BIOBADASER were diagnosed with RA (*n *= 4,006; 68.46%). SpA accounts for 26.04% (*n *= 1,524), AS 43% (*n *= 657), and PsA 37% (*n *= 570). The characteristics of patients are summarised in Table 1.

Compared with patients with RA, patients with SpA were younger (*p *< 0.001) and more frequently male (*p *< 0.001). Adalimumab was rarely used in SpA (15 treatments [0.90%] versus 577 [12.72%] in RA; *p *< 0.001).

The total exposure to TNF antagonists was 2,430 patient-years for SpA and 7,865 for RA. A detailed exposure rate by biologic and diagnosis is shown in Table 2. As expected, patients with RA not only had larger exposure in terms of patient-years, but also had used different biologic treatments more frequently. Adalimumab contributed little to the exposure to biologics in our population.

**Figure 1:**
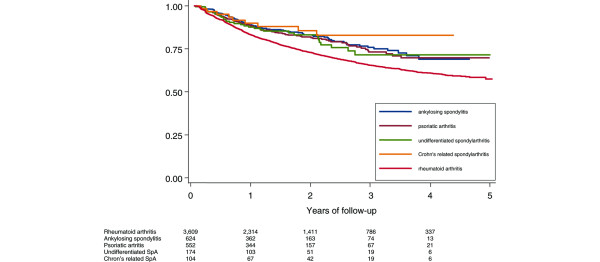
Survival of tumor necrosis factor antagonists in patients with spondylarthritis (SpA) and rheumatoid arthritis

Survival of TNF antagonists in SpA is significantly greater than in RA at 1, 2, and 3 years (Table 3), and the difference seems even larger with prolonged exposures (Figure 1). As shown in Table 3 and Figure 1, there are no significant differences in drug survival in the different types of SpA, although the group of "other SpA," which includes reactive arthritis, juvenile SpA, and chronic seronegative oligoarthritis, has lower drug survival (yet due to the small size of the group did not reach statistical significance [*n *= 42]).

The better survival in SpA is expressed by a hazard ratio (HR) for discontinuation of 0.66 (95% confidence interval [CI], 0.58–0.76) compared with RA. There are unevenly distributed factors in SpA and RA (Table 1) that had an impact on discontinuation: being older than 60 (HR = 1.21 [95% CI, 1.08–1.36]), being female (HR = 1.27 [95% CI, 1.13–1.43]), and using infliximab (HR = 1.53 [95% CI, 1.31–1.78]). After adjustment for these three factors, the HR for discontinuation in SpA was 0.66 [95% CI, 0.57–0.76] compared with RA. Survival of infliximab as first medication in SpA was significantly better than in RA (*p *= 0.0065). Similarly, survival of etanercept as first medication in SpA was significantly better than in RA (*p *= 0.0439). There were no differences however, when survival of infliximab or etanercept as second medication in RA was compared with their survival as second drug in SpA.

The distribution and the causes for discontinuation do not differ significantly in SpA and RA (*p*_chi-square _= 0.131). They are AE (45.4% versus 48.7%), lack of efficacy (34.6% versus 36.0%), and others (19.9% versus 15.3%). The type of infection was also distributed equally in SpA and RA. Herpes zoster virus and *Mycobacterium tuberculosis *were the leading identified microorganisms causing infection. Recommendations for screening and treatment of latent tuberculosis were put in place in March 2002 [35], and their impact has been reported elsewhere [37].

The HR for discontinuation due to AEs does not differ significantly among the types of SpA. On the contrary, the HR for discontinuation due to lack of efficacy is statistically greater for chronic seronegative arthritis than for the other types of SpA (HR = 6.7 [95% CI, 1.6–28.2]). Compared with patients with RA, patients with SpA treated with TNF antagonists have fewer AEs (17% versus 26%, respectively, had one or more AEs) that occur at a lower rate (13 events [95% CI, 11–14] in SpA versus 17 events per 1,000 patient-years in RA [95% CI, 16–18]). The incidence risk ratio_SpA versus RA _is 0.78 [95% CI, 0.68–0.89]). The HR of presenting an AE in SpA compared with RA is 0.69 [95% CI, 0.61–0.78]. However, as confirmed by the confidence intervals in Table 4, there were no differences in the rate of any particular AE. For the purpose of this study, discontinuation for AEs was considered when infusion or injection treatment after the event was not received within fourfold of the expected therapeutic interval. Under this definition, treatment was discontinued in 553 of 1,388 (39.8%) AEs in RA and in 123 of 329 (37.4%) in SpA (*p *= 0.412). Furthermore, the proportion of AEs that ended in hospitalisation or a prolonged hospital stay was 26.4% in RA and 21.3% in SpA (*p *= 0.056). Rate of AEs associated with death was 3.9% in RA and 1.5% in SpA (*p *= 0.034).

After adjustment for being older than 60 years (HR = 1.51 [95% CI, 1.37–1.67]), having a disease duration longer than three years prior to therapy with TNF antagonist (HR = 1.29 [95% CI, 1.12–1.48]), and using infliximab (HR = 2.52 [95% CI, 2.11–2.99]), the HR for an AE in SpA versus RA was 0.80 [95% CI, 0.70–0.91]. Adjustment for age as a continuous variable did not produce different results than adjustment for age as a dichotomized variable (older than 60 years).

## Discussion

In the present study, we have analysed the survival of TNF antagonists in patients with SpA in clinical practice in comparison with RA patients in a registry. We have found that, due in part to the occurrence of fewer AEs, patients with SpA have a 33% lower probability than patients with RA to discontinue TNF antagonists.

Measuring effectiveness of drugs in registries such as BIOBADASER is exposed to limitations. The voluntary medical registries need to be of high quality to be reliable [38]. The quality of our database was ensured by repeated external audits of the participating centres, as reported in Materials and methods. Following these quality assessment interventions, we expect an error of approximately 11% in the information considered relevant to the analysis. This is within the 0%-17% range reported by others [39].

Survival of a drug could be taken as an indication of drug effectiveness in the clinical setting. Furthermore, studies with extended follow-up and large numbers of patients are common in rheumatology in assessing long-term effectiveness of treatments [40]. However, these studies face limitations such as confounding by indication, patient selection, and the absence of a washout period [41].

Until recently, AS and PsA were difficult to evaluate in therapeutic trials because of the lack of standardised, widely accepted, or validated instruments for assessing disease and clinical response. This has changed in the past few years [42,43]. Although the pathogeneses of SpA have not been fully recognised, there appears to be a significant expression of TNF in the entheses and peripheral joints in all the conditions [44-47]. This has led to the use of TNF antagonists in the treatment of SpA resistant to NSAIDs and the traditional DMARDs. However, long-term studies in patients with SpA treated with TNF antagonists are not yet available, and optimal duration of therapy has not been established. In routine clinical practice, physicians tend to treat patients with SpA over protracted periods (as in RA) and, as such, these insights from our BIOBADASER database can provide valuable information about physicians' prescribing patterns. Of note is the better survival of TNF antagonists in patients with SpA than in patients with RA.

A limitation of our study is that measures of joint inflammation and damage were not collected, and therefore we can neither adjust for baseline severity nor measure improvement in each group. However, had we measured these parameters, it would be difficult to compare activity in RA with activity in PsA. Several parameters other than efficacy and safety (such as co-morbidity, costs, availability of other therapies, patient and physician expectations, and medication compliance) have an impact on the rate of discontinuation of a drug [48]. Absence of alternative medications in the treatment of SpA may have contributed to the favourable retention rate compared with RA. Another explanation could be the lower rate of AEs in SpA. This is explained, in part, by younger age and fewer co-medications in SpA than in RA, because age and co-medications are associated with a greater number of AEs [49]. Nevertheless, the difference in risk of AEs cannot be fully explained by these factors, because after the proper adjustments there is still a 20% lower risk in SpA compared with RA. Adjustment for potential confounders did not change the overall HR for discontinuation of SpA. Because all confounders were more likely to be present in RA, this suggests an interaction of one of the variables and the type of disease. We cannot prove that patients with SpA have fewer co-morbidities and use fewer concomitant medications than do patients with RA, because these data were collected only in patients who had AEs. However, comparison of both co-morbidities and concomitant medication in SpA and in RA patients who had had an AE reveals a significant difference (Mann-Whitney *U *test, *p *< 0.001). Also, RA itself could increase the rate of AEs in patients treated with TNF antagonists. Interestingly, the higher doses of infliximab recommended for SpA did not increase the rate of AEs. Although the dose was not actually noted, the recommended dose is 5 mg/kg for SpA and 3 mg/kg for RA (Summary of Product Characteristics of infliximab). Neither was it recorded whether the interval between infusions was shortened in patients not responding. This might have been the case in the early phases, when only infliximab was available, but was less probable thereafter.

Of note is that the HR of discontinuation of PsA versus RA was 0.81 (95% CI, 0.66–0.99). After adjustment for age, it lost its statistical significance. It may be that the symptoms of patients with PsA who predominantly have small-joint peripheral disease (that is, who appear to have RA) behave more like those of RA. This cannot be fully substantiated, because clinical data to assess the type of PsA were not included in the registry.

In patients with SpA, the rate of AEs with infliximab was greater than with etanercept. This could merely reflect an over-representation of patients treated with infliximab and with a longer follow-up. Furthermore, a bias toward the use of a "newer better" drug in the most severe cases or in non-responder patients)[50] may have distorted the observed efficacy; the availability of etanercept may have led to early discontinuation of infliximab, which had been cleared for use 40 months before etanercept. Head-to-head comparison will be needed to answer this question.

## Conclusion

Survival of TNF antagonists in SpA is better than in RA. At present, TNF antagonists are an optimal, safe, and effective therapeutic option for patients with severe SpA who fail to respond to traditional drugs. Because chronic therapy is necessary, continual review of this issue is necessary.

## Abbreviations

AE = adverse event; AS = ankylosing spondylitis; BASDAI = bath ankylosing spondylitis disease activity index; CI = confidence interval; DMARD = disease-modifying antirheumatic drug; HR = hazard ratio; NSAID = non-steroidal anti-inflammatory drug; PsA = psoriatic arthritis; RA = rheumatoid arthritis; RAE = relevant adverse event; SER = Spanish Society of Rheumatology; SpA = spondylarthritis; TNF = tumor necrosis factor.

## Competing interests

JJGR is on Advisory Boards of Schering-Plough, Wyeth, and Roche and has received lecture fees from Abbott Laboratories, Wyeth, and Schering-Plough.

## Authors' contributions

Both LC and JJGR were the designers of the study. JJGR prepared the manuscript, which was reviewed and modified by LC. LC planned and ran the analyses. The BIOBADASER Study group collected and checked the data without receiving any economic reward. Both authors read and approved the final manuscript.

## Figures and Tables

**Table 1:** Characteristics of patients with spondylarthritis and rheumatoid arthritis in BIOBADASER and medications used by diagnosis

					Total treatments with biologics, *n *(percentage) by diagnosis		
					
	*n *(percentage) by diagnosis in BIOBADASER	Age*	*n *(percentage), women	Disease duration*	Infliximab	Etanercept	Adalimumab
Rheumatoid arthritis	4,006 (68.5)	54 ± 13	3,166 (79.0)	10 ± 7	2,789 (60.6)	1,170 (25.4)	577 (12.5)
All spondylarthritis	1,524 (26.0)	45 ± 12^†^	537 (35.2)^†^	10 ± 8	1,180 (70.7)	472 (28.3)	15 (0.9)^†^
Ankylosing spondylitis	657 (11.2)	45 ± 12	152 (23.1)	12 ± 8	570 (80.5)	136 (19.2)	2 (0.3)
Psoriatic arthritis	570 (9.7)	47 ± 12	270 (47.4)	9 ± 7	347 (54.5)	281 (44.1)	9 (1.4)
Undifferentiated spondylarthritis	187 (3.2)	43 ± 12	70 (37.4)	8 ± 7	162 (79.0)	41 (20.0)	2 (1.0)
Crohn's disease arthritis	68 (1.2)	38 ± 13	32 (47.1)	7 ± 8	68 (94.4)	2 (2.8)	1 (1.4)
Juvenile spondylarthritis	19 (0.3)	30 ± 12	7 (36.8)	17 ± 14	17 (89.5)	2 (10.5)	-
Reactive arthritis	12 (0.2)	49 ± 9	2 (16.7)	8 ± 7	10 (83.3)	2 (16.7)	-
Chronic seronegative oligoarthritis	11 (0.2)	35 ± 9	4 (36.4)	4 ± 3	6 (40.0)	8 (53.3)	1 (6.7)

*In years, mean ± standard deviation

^†^*p *< 0.001 of the difference between rheumatoid arthritis and spondylarthritis as a group.

**Table 2:** Exposure to the different biologic response modifiers depending on diagnosis in BIOBADASER

Diagnosis	Infliximab	Etanercept	Adalimumab
Rheumatoid arthritis	5,521 (840)	1,724 (188)	526 (67)
All spondylarthritis	1,915 (211)	507 (35)	5.7 (3)
Ankylosing spondylitis	873 (87)	134 (13)	0.8 (0)
Psoriatic arthritis	636 (84)	325 (16)	2.6 (2)
Undifferentiated spondylarthritis	257 (28)	35 (4)	1.4 (0)
Crohn's related spondylarthritis	124 (9)	2.8 (0)	0.7 (0)
Other spondylarthritis	22 (3)	9.5 (2)	0.3 (1)

Numbers indicate total patient-years. In parentheses are the numbers of treatments discontinued.

**Table 3:** Drug survival at one, two, and three years by diagnosis

	Drug survival [95% confidence interval]		
	
Diagnosis	One year	Two years	Three years
Rheumatoid arthritis	0.83 [0.81–0.84]	0.72 [0.71–0.74]	0.65 [0.63–0.67]
All spondylarthritis	0.88 [0.86–0.90]*	0.82 [0.79–0.84]*	0.74 [0.71–0.78]*
Ankylosing spondylitis	0.88 [0.85–0.91]	0.82 [0.78–0.86]	0.76 [0.70–0.80]
Psoriatic arthritis	0.88 [0.84–0.90]	0.81 [0.77–0.84]	0.73 [0.67–0.78]
Undifferentiated spondylarthritis	0.88 [0.81–0.91]	0.83 [0.76–0.88]	0.72 [0.60–0.80]
Crohn's related spondylarthritis	0.90 [0.79–0.95]	0.86 [0.73–0.93]	0.82 [0.69–0.91]
Other spondylarthritis	0.79 [0.53–0.92]	0.61 [0.31–0.81]	0.61 [0.31–0.81]

*p of the difference versus survival at the same time in rheumatoid arthritis < 0.001 (by log-rank test).

**Table 4:** Types of adverse events occurring in patients with spondylarthritis and rheumatoid arthritis treated with biologics

	Spondylarthritis						Rheumatoid arthritis					
		
Biologic and total exposure	Infliximab 1,915		Etanercept 507		Adalimumab 5.7		Infliximab 5,521		Etanercept 1,724		Adalimumab 526	
					
Type of adverse event	Cases	IR [95% CI]	Cases	IR [95% CI]	Cases	IR [95% CI]	Cases	IR [95% CI]	Cases	IR [95% CI]	Cases	IR [95% CI]
					
Infusion reaction	72	4.14 [3.29–5.22]	1	0.20 [0.03–1.4]	0	-	252	5.09 [4.50–5.76]	7	0.43 [0.21–0.91]	6	1.36 [0.61–3.03]
Infection	102	5.87 [4.83–7.13]	5	1.01 [0.42–2.43]	1	18.90 [2.66–134.14]	350	7.07 [6.37–7.85]	28	1.74 [1.20–2.52]	56	12.71 [9.78–16.52]
Cutaneous	22	1.27 [0.83–1.92]	2	0.40 [0.10–1.61]	1	18.90 [2.66–134.14]	50	1.01 [0.77–1.33]	21	1.30 [0.85–2.00]	40	9.08 [6.66–12.38]
Cytopenia	2	0.12 [0.03–0.46]	0	-	0	-	27	0.54 [0.37–0.80]	3	0.19 [0.06–0.58]	3	0.68 [0.22–2.11]
Neoplastic	6	0.35 [0.16–0.77]	1	0.20 [0.03–1.4]	0	-	20	0.40 [0.26–0.63]	8	0.50 [0.25–0.99]	1	0.23 [0.03–1.61]
Pulmonary	4	0.23 [0.09–0.61]	0	-	0	-	21	0.42 [0.28–0.65]	4	0.25 [0.09–0.66]	3	0.68 [0.22–2.11]
Cardiovascular	13	0.75 [0.43–1.29]	3	0.61 [0.20–1.88]	0	-	64	1.29 [1.01–1.65]	8	0.50 [0.25–0.99]	13	2.95 [1.71–5.08]
Endocrine	1	0.06 [0.01–0.41]	0	-	0	-	5	0.10 [0.04–0.24]	0	-	0	-
Gastrointestinal	25	1.44 [0.97–2.13]	3	0.61 [0.20–1.88]	0	-	32	0.64 [0.46–0.91]	5	0.31 [0.13–0.75]	19	4.31 [2.75–6.76]
Ophthalmologic	4	0.23 [0.09–0.61]	0	-	0	-	2	0.04 [0.01–0.16]	3	0.19 [0.06–0.58]	2	0.45 [0.11–1.82]
Psychiatric	3	0.17 [0.06–0.54]	0	-	0	-	2	0.04 [0.01–0.16]	0	-	6	1.36 [0.61–3.03]
Neurological	5	0.29 [0.12–0.69]	1	0.20 [0.03–1.43]	0	-	15	0.30 [0.18–0.50]	1	0.06 [0.01–0.44]	8	1.82 [0.91–3.63]
Gynecological	0	-	0	-	0	-	3	0.06 [0.02–0.19]	0	-	1	0.23 [0.03–1.61]
Urological	4	0.23 [0.09–0.61]	0	-	0	-	4	0.08 [0.03–0.22]	1	0.06 [0.01–0.44]	3	0.68 [0.22–2.11]
Others	17	Not estimated	2	Not estimated	0	Not estimated	83	Not estimated	10	Not estimated	23	Not estimated

Data are presented as cases documented and as incidence rate (IR) and 95% confidence interval (CI) per 100 patient-years of exposure.
